# A Scarless Healing Tale: Comparing Homeostasis and Wound Healing of Oral Mucosa With Skin and Oesophagus

**DOI:** 10.3389/fcell.2021.682143

**Published:** 2021-07-26

**Authors:** Diana Pereira, Inês Sequeira

**Affiliations:** Institute of Dentistry, Barts and the London School of Medicine and Dentistry, Queen Mary University of London, London, United Kingdom

**Keywords:** oral mucosa, oesophagus, skin, homeostasis, wound repair, regenerative therapy, tissue engineering, regenerative medicine

## Abstract

Epithelial tissues are the most rapidly dividing tissues in the body, holding a natural ability for renewal and regeneration. This ability is crucial for survival as epithelia are essential to provide the ultimate barrier against the external environment, protecting the underlying tissues. Tissue stem and progenitor cells are responsible for self-renewal and repair during homeostasis and following injury. Upon wounding, epithelial tissues undergo different phases of haemostasis, inflammation, proliferation and remodelling, often resulting in fibrosis and scarring. In this review, we explore the phenotypic differences between the skin, the oesophagus and the oral mucosa. We discuss the plasticity of these epithelial stem cells and contribution of different fibroblast subpopulations for tissue regeneration and wound healing. While these epithelial tissues share global mechanisms of stem cell behaviour for tissue renewal and regeneration, the oral mucosa is known for its outstanding healing potential with minimal scarring. We aim to provide an updated review of recent studies that combined cell therapy with bioengineering exporting the unique scarless properties of the oral mucosa to improve skin and oesophageal wound healing and to reduce fibrotic tissue formation. These advances open new avenues toward the ultimate goal of achieving scarless wound healing.

## Introduction

Epithelial tissues provide the body’s first line of protection from physical, chemical and biological damage. Mammalian epithelia vary in structure throughout the body according to their function and microenvironment. Skin is considered the largest organ of our body; however, it is not the only epithelium exposed to the external environment. The airways, digestive tract, as well as the urinary and reproductive systems, are all exposed to external stress and are lined by an epithelium, sharing some important structural and functional features.

In this review, we focus on three stratified squamous epithelial tissues – the skin, the oesophagus and the oral mucosa – and provide a comparative analysis of the architecture, cell composition and behaviour of these three different tissues during homeostasis and wound healing. We discuss the outstanding regenerative potential of the oral mucosa and how its scarless wound healing properties can be applied to the other tissues.

## Comparative Analysis of Skin, Oesophagus, and Oral Mucosa

**FIGURE 1:**
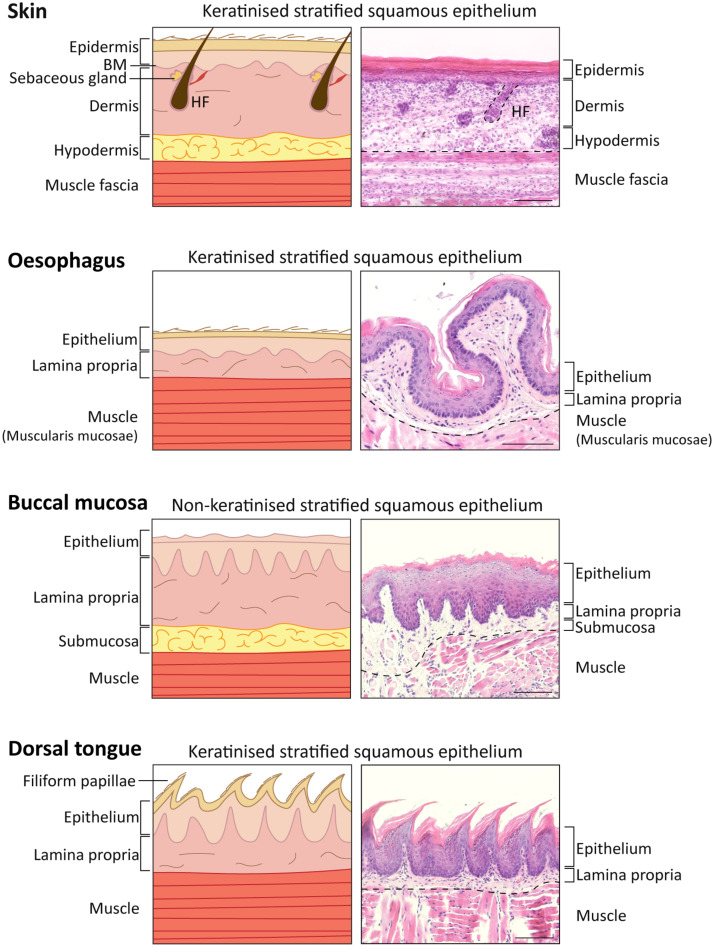
Comparison of skin, oesophagus, oral mucosal tissue structure in *Mus musculus*. Diagram **(Left)** and representative histological images **(Right)** of skin, oesophagus and oral mucosa keratinised (dorsal tongue) and non-keratinised (buccal mucosa) tissues identifying the different layers. 5 μm-sections collected from a 16-week-old C57BL/6 mouse stained with haematoxylin and eosin staining (H&E). Scale bar: 100 μm. HF, hair follicle.

**FIGURE 2:**
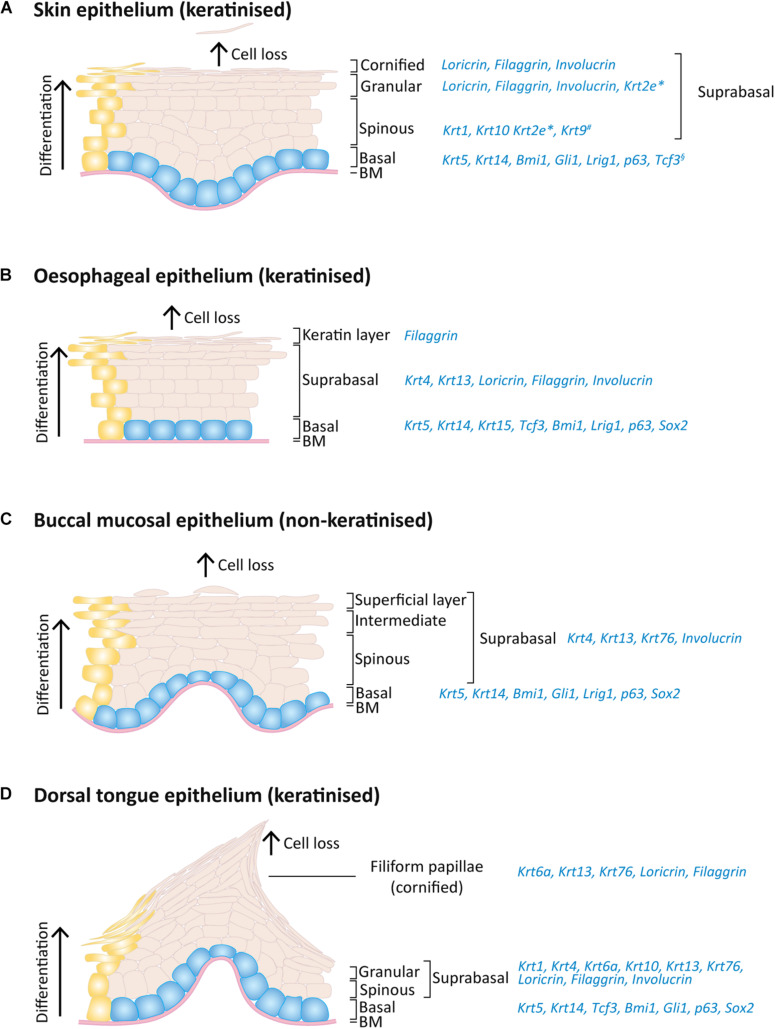
Expression pattern of keratins and others markers on the adult mouse skin, oesophagus and oral epithelia. Schematic of epithelial layers and respective expression markers for **(A)** skin, **(B)** oesophageal, **(C)** buccal mucosa, and **(D)** dorsal tongue epithelia. During normal epithelial homeostasis, epithelial cells proliferate on the basal layer **(blue)** and keratinocyte differentiation **(yellow)** is accompanied by an upward migration through the suprabasal layers, replacing dead cells that shed from the epithelium surface. *Expressed only on ear, sole and tail skin; ^#^expressed only on sole and palm skin; ^§^expressed only on paw skin.

Adult epithelia harbour resident stem cells (SCs) responsible for homeostasis and tissue repair. The epithelial lining of the skin develops from the ectoderm, the oesophageal epithelium derives from the endoderm, while the oral epithelium derives both from ectoderm and endoderm (Wells and Melton, 1999; Fuchs, 2007; Que et al., 2007; Rothova et al., 2012). Skin, oesophagus and oral mucosa share global cellular architecture (Figure 1) and homeostasis, however several studies have highlighted different markers for their SCs and differentiated cells (Figure 2).

### Skin, Oesophagus and Oral Mucosa Structural Comparison

The skin is comprised of three different layers: epidermis, dermis and hypodermis, and harbours additional appendages, such as hair follicles, nails, sweat and sebaceous glands (Watt, 2014). The interfollicular epidermis (IFE) is the outermost layer and is responsible not only for mechanical protection from the hostile environment, but also prevents from dehydration and invasion by microorganisms. The IFE is a multi-layered stratified squamous epithelium with four layers that have different degrees of differentiation: basal, spinous, granular and stratum corneum or cornified layer (Figures 1, 2A), and is composed of keratinocytes, Merkel cells, melanocytes, Langerhans cells and lymphocytes (Rushmer et al., 1966; Matoltsy, 1986; Odland, 1991; Holbrook, 1994; Joost et al., 2020). The IFE is separated from the underlying dermis by a basement membrane (Figure 1), an extracellular matrix (ECM) rich in type IV collagen and laminin (Timpl and Brown, 1996).

The dermis is the connective tissue layer that provides skin elasticity and tensile strength (Frantz et al., 2010) and it is mainly composed of fibroblasts, but also monocytes, macrophages, mast cells, lymphocytes, dermal adipocytes, as well as blood vessel- and sensory nerves-related cells (Lai-Cheong and McGrath, 2013). Using intra-vital imaging on normal mouse ear and paw skin showed that fibroblasts maintain a stable position, and that upon loss of neighbouring cells, the cell membranes extend to fill in the space in a Rac1-dependent process. This process is also conserved upon fibroblast loss in skin ageing (Marsh et al., 2018). However, it is the non-cellular component of the dermis – the ECM – that provides the scaffolding for the skin cellular constituents and that regulates the signalling required for tissue morphogenesis, differentiation and homeostasis (Sorrell and Caplan, 2004; Frantz et al., 2010).

The dermis can be separated into three spatially distinct layers with unique characteristics in development, regeneration and fibrosis: (1) the papillary layer, closest to the epidermis with a high cell density and loose connective tissue and expressing CD90^+^CD39^+^FAP^+^ in human; (2) the reticular layer, with lower cell density but rich in connective tissue and expressing FAP^–^CD90^+^ in human (and CD36^+^ for the lower reticular); and (3) the hypodermis which consists mainly of adipose tissue, loose connective tissue and is highly vascularised and rich in hormones and growth factors and expressing CD90^+^CD36^+^ in human (Figure 1; Harper and Grove, 1979; Azzarone and Macieira-Coelho, 1982; Schafer et al., 1985; Sorrell et al., 1996; Freinkel and Woodley, 2001; Sorrell and Caplan, 2004; Watt and Fujiwara, 2011; Driskell et al., 2013; Driskell and Watt, 2015; Sriram et al., 2015; Hiraoka et al., 2016; Philippeos et al., 2018; Korosec et al., 2019). Another fibroblast subpopulation associated with hair follicles lies in the dermal papilla and on the hair follicle dermal sheath, and belongs to the papillary lineage (Reynolds and Jahoda, 1991; Jahoda and Reynolds, 1996; Driskell et al., 2013; Joost et al., 2020). Several studies have highlighted the functional heterogeneity of fibroblasts with different healing potential (Driskell et al., 2013; Rinkevich et al., 2015; Mastrogiannaki et al., 2016; Jiang and Rinkevich, 2018; Jiang et al., 2018; Philippeos et al., 2018; Tabib et al., 2018; Correa-Gallegos et al., 2019; Guerrero-Juarez et al., 2019; Abbasi et al., 2020; Joost et al., 2020; Phan et al., 2021) as well as differences in the expression of collagen subtypes and proteoglycans (Meigel et al., 1977; Zimmermann et al., 1994; Sorrell et al., 1999; Sorrell and Caplan, 2004) and response to different signals originating from neoplastic epidermal SCs (Lichtenberger et al., 2016). Papillary fibroblasts are more proliferative than site-matched reticular fibroblasts, in both mouse and human skin (Harper and Grove, 1979; Azzarone and Macieira-Coelho, 1982; Schafer et al., 1985; Sorrell et al., 1996; Sorrell and Caplan, 2004) and more effectively support the formation of a multi-layered epithelium in two- and three-dimensional (3D) cultures (Higgins et al., 2017; Korosec and Lichtenberger, 2018; Philippeos et al., 2018). Reticular dermis is richer in fibrous connective tissue and, when in culture, reticular dermal fibroblasts contract collagen latices faster than papillary dermal fibroblasts (Schafer et al., 1985; Sorrell et al., 1996). According to lineage tracing and skin reconstitution assays in mice, reticular fibroblasts descending from PDGFRα^+^Dlk1^+^ progenitors are responsible for the first wave of dermal wound repair and produce the bulk of the ECM whereas papillary fibroblast lineage supports healthy skin regeneration and hair follicle development, namely through expression of the key transcription factor Lef1 (Driskell et al., 2013; Rognoni et al., 2016, 2018; Phan et al., 2020). More recently, the quiescence-associated factor hypermethylated in cancer 1 positive (Hic1^+^) progenitors, primarily distributed in the reticular dermis, was shown to robustly contribute to regenerate injured dermis and to populate neogenic hair follicles in adult mice (Abbasi et al., 2020). As for the hypodermis, the deepest layer of the mammalian skin that provides insulation and cushioning, is crucial for wound healing, re-epithelialisation and angiogenesis processes (Freinkel and Woodley, 2001; Rivera-Gonzalez et al., 2014; López et al., 2018; Zomer et al., 2020).

A recent study has identified an additional fibroblast subpopulation below the hypodermis called the fascia that contribute to skin scar formation (see section “The Outstanding Regenerative Potential of Oral Mucosa – Scarless Wound Healing”; Correa-Gallegos et al., 2019; Jiang et al., 2020a; Jiang and Rinkevich, 2021).

In continuity with the skin epithelium, the stratified oral mucosa provides an important barrier to the external challenges. The structure of the oral epithelium comprises a stratified squamous epithelium (keratinised or non-keratinised) and the underlying lamina propria, which is rich in connective tissue, fibroblasts, nerves, minor salivary glands and blood vessels (Jones and Klein, 2013; Hand and Frank, 2014; Figure 1).

The non-keratinised oral epithelia comprise basal, spinous, intermediate and superficial layers, while the keratinised oral epithelia resemble the skin epidermis and include basal, spinous, granular and cornified layers (example of keratinised *vs.* non-keratinised oral epithelia in Figures 1, 2; comparison between all mouse oral epithelia reviewed in Jones and Klein, 2013). Furthermore, the oral mucosa is subdivided in masticatory (hard palate and gingiva), specialised (dorsal tongue) and lining subtypes (soft palate, buccal mucosa, ventral tongue, intra-oral lips and alveolar mucosa) (Gartner, 1991; Jones and Klein, 2013), reflecting the different structures within the oral cavity. For instance, the cheek buccal mucosa and soft palate are covered by non-keratinised lining mucosa which confers flexibility (Figure 1). The hard palate and gingiva are characterised by a keratinised masticatory epithelium prepared for stresses caused by chewing food. The tongue presents two different phenotypes: the ventral surface displays a non-keratinised lining epithelium, and the dorsal surface is covered by a specialised keratinised epithelium (Figures 1, 2; Jones and Klein, 2013; Hand and Frank, 2014; Groeger and Meyle, 2019). The specialised epithelium of the dorsal tongue houses four types of lingual papillae, three gustatory papillae (fungiform, circumvallate and foliate) with taste buds for sensorial stimuli, and filiform papillae important to grip and process food (Mistretta and Kumari, 2017). Filiform papillae are found in large numbers through the dorsal tongue and present a spinous cone-shaped structure (Figures 1, 2D; Hume and Potten, 1976).

Oral (gingival) fibroblasts are known to resemble foetal skin regenerative potential, namely on their migratory capacity through production of MSF, a migration stimulating factor not present in adult skin (Irwin et al., 1994).

From a development perspective, dorsal skin and oral mucosal fibroblasts have different origins: while the non-cranial dorsal skin dermis has an *Engrailed1*-lineage-positive somitic origin, the oral mucosa lamina propria and cranial skin dermis originates from *Wnt1*-lineage-positive neural crest cells (Janebodin et al., 2011; Ishii et al., 2012; Rinkevich et al., 2015). This may be the basis for the intrinsic phenotypic differences between oral and skin fibroblasts in wound healing. For instance, CD90^+^CD26^+^ skin fibroblasts were linked to scarring in skin wound healing, however, in gingiva CD26^+^ fibroblasts are only residually present (Mah et al., 2017; Worthen et al., 2020). Oral mucosal fibroblasts are also primed with higher expression levels of hepatocyte growth factor and its most relevant isoform NK1, therefore more effectively resist to TGF-β1-driven myofibroblast differentiation when compared to dermal fibroblasts (Dally et al., 2017). Another crucial difference relies on the phenotypic activity of the matrix metalloproteinase (MMP) tissue inhibitors (TIMP), namely TIMP-1 and TIMP-2 production, which in the oral mucosa is reduced, therefore allowing for increased MMP-2 activity in the remodelling phase of oral wound healing (Stephens et al., 2001).

Importantly, the epithelial-stromal interaction is key determinant of the phenotypic dynamics of the epithelium in homeostasis and when challenged. The epithelium is affected by the underlying mesenchymal cells, as these produce keratinocyte growth factor and hepatocyte growth factor/scatter factor molecules, important for the regulation of epithelial growth and integrity (Grøn et al., 2002; Costea et al., 2003; McKeown et al., 2003; Shannon et al., 2006; Sa et al., 2019). Furthermore, the epithelial-stromal-immune cell crosstalk in gingival mucosa was recently described as determinant of inducing an immune response to environmental cues and in regulating mucosal immunity (Nowarski et al., 2017; Caetano et al., 2021; Williams et al., 2021).

The submucosal layer of the oral cavity can be compared to the hypodermis in skin, being composed of loose fatty or glandular connective tissue. The presence of a submucosal layer depends on the oral cavity region and is directly linked to the flexibility of the attachment of the oral mucosa to underlying structures. In regions of lining epithelium (such as the cheek buccal mucosa, lips and some hard palate regions) this layer separates the oral mucosa from the bone or muscle below (Figure 1), while regions of masticatory and specialised mucosa (such as gingiva and some hard palate regions) lack this layer (Squier and Kremer, 2001).

Compared to skin and oral mucosa, the oesophagus epithelium is relatively simpler. Given its physiological function of transferring food from the oral cavity to the stomach, this organ is extended from the upper to the lower oesophageal sphincters which are respectively overlapped by the pharyngoesophageal and gastroesophageal junctions. The sphincters open during swallowing and the oesophagus initiates the process of peristalsis to assure the unidirectional transport of the content to the stomach. The mouse oesophagus comprises a keratinised stratified squamous epithelium, differing from non-keratinised human oesophageal epithelium (Figure 1). There are a few other key aspects that differentiate the mouse and human oesophageal epithelia. In humans, the oesophageal epithelium is folded around structures called papillae, which separates the basal layer into either interpapillary or papillary basal layers; it is also characterised by the presence of submucosal glands. This contrasts with the simple epithelium found in mice, devoid of papillae and glands (Messier and Leblond, 1960; Seery, 2002; Doupé et al., 2012; Alcolea, 2017). Additionally, while in mice the oesophageal epithelium comprises a basal layer of proliferating cells (Goetsch, 1910; Messier and Leblond, 1960; Marques-Pereira and Leblond, 1965; Gavaghan, 1999), in humans, cycling cells extend to the 5^*th*^-6^*th*^ suprabasal layers (Barbera et al., 2015).

The oesophagus mucosa is composed of two other layers: the lamina propria, which in this organ is a very thin layer of connective tissue supporting the epithelium, as well as a thin layer of longitudinally organised smooth muscle (Goetsch, 1910; Oezcelik and DeMeester, 2011). To add to this diversity, it is known that the human oesophagus is not only composed of squamous epithelium, but on the most distal area there is a 1-2cm transition to columnar epithelium, which is the same lining epithelium covering the stomach (Gavaghan, 1999). Furthermore, the muscularis mucosae thickness increases from the most proximal to the most distal part of the oesophagus (Goetsch, 1910; Oezcelik and DeMeester, 2011).

Both the oral and the oesophageal epithelia are devoid of appendages. Although they belong to the gastrointestinal tract, they share the same stratified epithelium architecture as the skin rather than the single layer of cells that line the stomach, the small intestine and the large intestine, important for greater absorption capacity (Goetsch, 1910; Gordon, 1994).

### Comparison of the Keratin Expression Programme Between Different Epithelia

Keratins are intermediate filament proteins of epithelial cells providing mechanical integrity and structure to the epithelia and act as a scaffold that enables cells to resist stress and damage, which is essential for normal tissue function (Coulombe et al., 1991; Moll et al., 2008). Changes in keratin synthesis leads to alterations in cell movement or cell differentiation and, consequently, their function (Vassar et al., 1991; Singh and Gupta, 1994). Mutations that impair keratin assembly have been identified in a range of human skin or multifactorial disorders, such as epidermolysis bullosa, typically leading to loss of epithelial integrity, abnormal differentiation and affecting epithelial regeneration (Lane, 1994; Quinlan et al., 1994; Knöbel et al., 2015; Herrmann and Aebi, 2016; Bardhan et al., 2020).

While different epithelia exhibit different patterns of keratin expression (Franke et al., 1981) the keratin patterns are similar between the same anatomic regions of different species. During epithelial homeostasis, epithelial cells migrate from the basal into the suprabasal layer and progressively loose their proliferative potential and begin to synthesise a set of structural proteins (Candi et al., 2005). The switch in the keratin expression from proliferating basal cells to differentiated suprabasal cells indicates a change in the cell cytoskeleton organisation, influencing their functional properties.

In all skin, oesophageal and oral stratified squamous epithelia, the basal dividing cells produce keratin 5 (Krt5) and Krt14 (Squier and Kremer, 2001; Rosekrans et al., 2015; Gonzales and Fuchs, 2017). Krt15 is additionally expressed in the oesophageal basal cells (Rosekrans et al., 2015; Giroux et al., 2017). As cells leave the basal layer and start differentiating, the keratin expression suffers a transition to other keratins and differences arise between types of epithelia. For instance, mouse skin epidermal suprabasal cells switch to expressing Krt10 and Krt1 on interfollicular epidermis and Krt2e on the ears, soles and tail (Fuchs and Green, 1980; Candi et al., 2005; Fischer et al., 2016; Figure 2A). Interestingly, the epidermis of palms and soles, which are the thickest epidermis withstanding the highest degree of mechanical stress the body is exposed to, also express Krt9 in suprabasal layers to provide additional mechanical reinforcement (Knapp et al., 1986; Moll et al., 1987; Candi et al., 2005; Fu et al., 2014).

The heterogeneity of oral epithelia is reflected in its suprabasal keratin expression. The non-keratinised lining mucosa shares the expression of Krt4 and Krt13 (Dale et al., 1990), whereas the palatal and gingival masticatory epithelia are keratinised and share the expression of Krt1, Krt2p (now called Krt76) and Krt10 with skin (Dale et al., 1990; Collin et al., 1992). The gingiva itself is composed of a heterogeneous combination of keratin expression varying between the gingival epithelium mentioned above, the sulcular epithelium (expressing Krt4 and Krt13) and junctional epithelium (expressing Krt8, Krt13, Krt16, Krt18 and Krt19) (Dale et al., 1990; Groeger and Meyle, 2019). Regarding the specialised epithelium of the dorsal tongue, a heterogeneous pattern is also found: Krt4 and Krt13 are expressed in the interpapillary zone and anterior papillae, Krt1 and Krt6a are expressed in the anterior papillae and Krt1 and Krt10 are locally expressed in the posterior papillae (Dale et al., 1990; Howard et al., 2014; Nishiguchi et al., 2016). A recent study has also shown the expression of Krt76 in the palate, buccal mucosa and dorsal tongue suprabasal layers, including filiform papillae (Figures 2C,D; Sequeira et al., 2018).

With more similarities with oral than with skin epithelia, the oesophageal epithelium expresses Krt4 and Krt13 on the suprabasal layers (Figure 2B; Treuting et al., 2012; Rosekrans et al., 2015; Zhang et al., 2017). The mouse oesophagus contains an acellular layer of keratin on the top of the squamous epithelium, similar to the skin, however this keratin layer is absent in the human oesophagus (Treuting et al., 2012).

In addition to keratins, important transcription factors are also expressed in the basal layer of different stratified epithelia: Lef/Tcf-family transcription factor Tcf3 was found in paw skin, dorsal tongue and oesophagus (Howard et al., 2014); Bmi1, Lrig1 and p63 are enriched as well in all these epithelial basal layers (Figure 2; Que et al., 2007; Senoo et al., 2007; Choy et al., 2012; Jones and Klein, 2013; Zhang et al., 2017; Byrd et al., 2019; Jones et al., 2019; Piedrafita et al., 2020); Gli1^+^ cells are present in oral mucosa and skin epithelial basal layer while Sox2 is in oesophageal and oral epithelia, including tongue taste bud cells (Que et al., 2007; Jones and Klein, 2013; Zhang et al., 2017; Jones et al., 2019; Ohmoto et al., 2020; Figure 2).

The proteins filaggrin, involucrin, and loricrin are also expressed in the suprabasal layers of these epithelia, being key differentiation proteins involved in the thickening of the cornified cell envelope (Figure 2; Mehrel et al., 1990; Squier and Kremer, 2001; Howard et al., 2014; Nishiguchi et al., 2016; Quiroz et al., 2020). Considering the lack a cornified layer in non-keratinised epithelia, keratinocytes retain their nucleus and despite presenting membrane-coating granules, the accumulation and aggregation of cytokeratins with formation of bundles of filaments seen in keratinised epithelia is much less pronounced (Squier, 1977).

Interestingly, keratin expression programmes can change when epithelial cells are exposed to a different environment. Epithelial cells respond to extrinsic signals and change their identity when placed in a different microenvironment, as observed when oesophageal, thymic or cornea epithelial are placed on skin microenvironment (Ferraris et al., 2000; Bonfanti et al., 2010; Bejar et al., 2021). For instance, when oesophageal epithelial cells are grafted into skin, the suprabasal layers loose Krt4 expression as it transforms into a skin identity (Bejar et al., 2021). The mechanisms regulating this identity change remain to be elucidated.

### Epithelia Homeostasis and Cellular Differentiation

Tissues such as the squamous epithelia of the epidermis, oral cavity and oesophagus hold the natural capacity of self-renewal, with resident adult SCs actively replacing dying cells to accomplish homeostasis. The skin epidermis is by far the most studied epithelium, and this reflects the depth of the knowledge on SC behaviour and differentiation. In adult skin, different epithelia maintain homeostasis by their own pool of SC niches that are found in the basal layer of the IFE, as well as in the sweat glands, touch domes and hair follicle (Fuchs and Green, 1980; Cotsarelis et al., 1990; Blanpain et al., 2004; Morris et al., 2004; Ito et al., 2005; Legué and Nicolas, 2005; Clayton et al., 2007; Jaks et al., 2008; Jensen et al., 2009; Snippert et al., 2010b; Legué et al., 2012; Lu et al., 2012; Sequeira and Nicolas, 2012; Doucet et al., 2013; Page et al., 2013; Schepeler et al., 2014; Sada et al., 2016; Donati et al., 2017; Mesler et al., 2017; Yang et al., 2017).

**FIGURE 3:**
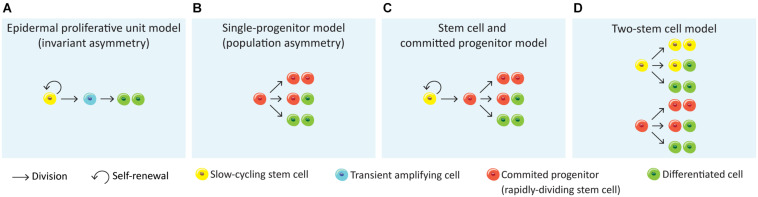
Models for epithelial self-renewal. **(A)** Epidermal proliferative unit or invariant asymmetry model (Potten, 1974) suggests that epithelial renewal relies on quiescent slow-cycling SCs that generate transient amplifying actively cells, which in turn generate non-dividing, differentiated cells. **(B)** A second model defends that the epithelial renewal is achieved long-term by a single population of actively cycling and through stochastic fate of committed progenitor cells that directly generate differentiated cells (single-progenitor model) (Clayton et al., 2007; Doupé et al., 2010). **(C)** The stem cell and committed progenitor model aroused from the observation of a fast-cycling stem cell population (committed progenitor) within the basal layer that is generated by slow-cycling stem cells. These progenitors eventually produce differentiated cells, however due to its short lifespan their contribution to wound healing is limited (Mascré et al., 2012; Sánchez-Danés et al., 2016). **(D)** The two stem cell model suggests the co-existence of two stem cell populations independent from each other, with different division rates (Joost et al., 2016; Rompolas et al., 2016; Sada et al., 2016; Aragona et al., 2020; Piedrafita et al., 2020).

There has been a great effort to understand the organisation and fate of stem cells in the basal layer that maintain tissue homeostasis. The first proposed model was the SC-transient amplifying cell hierarchy of the epidermal proliferative unit (EPU) (Potten, 1974; Figure 3A). The EPU model defends that each stack of cornified cells is maintained by a single slow-cycling SC basally located within the basal layer. The SC divides asymmetrically to generate another SC and a daughter transient amplifying cell, organised in 3D columns. The transient amplifying cells show high proliferative potential, undergo a fixed number of divisions prior upward migration and differentiation (Potten, 1974; Mackenzie, 1997). This model predicts clone size to rise into a plateau and then remain stable, although this was ruled out by lineage-tracing experiments (Clayton et al., 2007).

More recently, another study proposed the “population asymmetry” or “single-progenitor” model (Clayton et al., 2007), where the epidermal maintenance is achieved long-term through stochastic fate of a single committed keratinocyte progenitor in the basal layer from a pool of relatively fast cycling undifferentiated Krt5^+^Krt14^+^ epidermal SCs (Figure 3B). This pool is maintained by an autocrine mechanism of *Wnt* signalling (Lim et al., 2013). According to this model, SCs divide to generate one basal cell that attaches to the basement membrane and one committed progenitor cell which will be prone to leave the basal layer to enter an upward differentiation process. The progenitor population will continuously divide wile committed cells leave the basal layer and differentiate. This model suggests that the progenitor population randomly undergoes either asymmetrical or symmetrical divisions, the latter giving two progenitors or two differentiated cells (Clayton et al., 2007; Doupé et al., 2010; Lim et al., 2013; Rompolas et al., 2016; Figure 3B). Lineage-tracing experiments have shown that following this stochastic choice between symmetric or asymmetric SC division, the mean clone size progressively increases with time (Clayton et al., 2007).

An alternative to this model is the “Stem cell – committed progenitor model” (Figure 3C) that proposes a hierarchy of slow-cycling SCs that will give rise to active SCs (progenitors) which will then follow symmetric of asymmetric divisions to self-renew or to generate the differentiated cells (Mascré et al., 2012; Sánchez-Danés et al., 2016; Figure 3C).

Finally, a fourth model proposes the existence of two SC populations that differ in their proliferative dynamics, their gene-expression profile and their ability to repair the epidermis after injury (Figure 3D; Rompolas et al., 2016; Sada et al., 2016; Piedrafita et al., 2020). Some studies have already demonstrated heterogeneity within the mouse IFE basal cells. Joost and colleagues found two basal subpopulations in mouse dorsal IFE basal I and basal II, differing in the additional expression of *Avpi1*, *Krt16*, *Thbs1*, and the transcription factor *Bhlhe40* by IFE basal I (Joost et al., 2016). Furthermore, the IFE progenitors found in different regions of the body were slow-cycling cells able to both self-renew and give rise to intermediate progenitors with a shorter lifespan and greater tendency to differentiation (Mascré et al., 2012; Sada et al., 2016; Sánchez-Danés et al., 2016; Piedrafita et al., 2020). The different observations on IFE basal cell populations in relation to the anatomical position were proposed to be dependent on hair follicle density in those regions. Both the distance to the hair follicles and its cycling status were shown to influence clonal progression reflecting fast- and slow-cycling progenitors (Roy et al., 2016; Gonzales and Fuchs, 2017). This two-stem cell model (Figure 3D) was very recently reinforced by Aragona and colleagues through the study of cellular and molecular mechanisms underlying stretch-mediated expansion *in vivo* (Aragona et al., 2020). The authors show that stretching induces changes in the renewal activity of a subset of epidermal SCs, which is crucial for expansion, while a second progenitor subpopulation committed to differentiation is preserved. These events were shown to be more consistently governed by the two-stem cell model when compared to the single-progenitor model (Figures 3B,D). Interestingly, a recent single-cell RNA-sequencing analysis of human neonatal foreskin discovered four basal SC populations with differential spatial distribution on the rete ridges of the epidermis, agreeing with a model of multiple SC pools that differ in their proliferation capacity (Wang et al., 2020). Future lineage-tracing, single-cell and microscopic analysis will be needed to further elucidate the basal layer cellular heterogeneity as well as novel markers and regulators.

The hair follicle has separate pools of long-term SCs [CD34^+^ (Blanpain et al., 2004), Gata6^+^ (Donati et al., 2017), Lgr5^+^ (Jaks et al., 2008), Lgr6^+^ (Snippert et al., 2010a), Lrig1^+^ (Jensen et al., 2009)] that are responsible for the homeostasis and the cycling regeneration of the hair follicle; and some of these subpopulations can contribute to the IFE for wounding regeneration, although they do not contribute to normal homeostasis maintenance of the IFE (Ito et al., 2005; Legué et al., 2012; Mesa et al., 2015; Liakath-Ali et al., 2018; Dekoninck and Blanpain, 2019; Abbasi et al., 2020).

IFE and oesophageal epithelia appear to share common homeostasis mechanisms (Piedrafita et al., 2020). As for the IFE, oesophageal homeostasis mechanisms of cell behaviour remain controversial. Some studies postulated that a hierarchy of stem and transient amplifying cells maintains homeostasis. Croagh and colleagues reported the existence of three basal cell subpopulations, according to the expression profiles of α_6_integrin and transferrin receptor (CD71): one α_6_^*bri*^CD71^*dim*^ is a putative oesophageal SC population, the α_6_^*bri*^CD71^*bri*^ represents the transient amplifying cell population and the third population α_6_^*dim*^ which is a population of early differentiating cells (Croagh et al., 2007). In agreement to the postulated heterogeneity of basal cells, DeWard and colleagues used a combination of cell-surface markers and labelled proliferating basal epithelial cells *in vivo* to infer cell-cycle profiles and proliferation kinetics. Differences on the expression of α6 integrin (Itgα6, also known as CD49f) and β4 integrin (Itgβ4, CD104) in Sox2^+^ basal cells, combined with CD73 and Krt14, Krt13and Krt4 revealed three different basal subpopulations: Itgα6/Itgβ4^*High*^CD73^+^ is a SC population, the faster dividing Itgα6/Itgβ4^*High*^CD73^–^ is a transient-amplifying population and Itgα6/Itgβ4^*Low*^ represented the more differentiated basal cell population (DeWard et al., 2014). However, more studies argue that proliferation of a single progenitor population is confined to the basal layer in contact to the basement membrane and as progenitors are committed to differentiation, they withdraw from the cell cycle and migrate from this layer toward the epithelial surface. The fate of a dividing cell is randomly assigned, however the probabilities are balanced, so equal proportions of progenitor and differentiated cells are generated to maintain cellular homeostasis (Piedrafita et al., 2020). How this balance is maintained is not yet clear (Jankowski, 1993; Doupé et al., 2010, 2012; Alcolea et al., 2014; Frede et al., 2016). Recently, Giroux and colleagues defended the existence of a long-lived Krt15^+^ population with stem/progenitor cell characteristics through *in vivo* lineage-tracing and pointed against the single-progenitor model (Giroux et al., 2017).

All these paradigms around the proposed models of skin and oesophageal epithelia cell dynamics prompted Piedrafita and colleagues to conduct an in-depth study of nine lineage-tracing datasets in both oesophagus and various skin regions (paw, ear, back, tail scale and tail interscale) (Doupé et al., 2010, 2012; Mascré et al., 2012; Lim et al., 2013; Füllgrabe et al., 2015; Sada et al., 2016; Sánchez-Danés et al., 2016; Giroux et al., 2017; Murai et al., 2018), defending that divergent hypothesis result from distinct datasets analysis through distinct interpreting and suitable procedures, lacking alternative hypotheses tests. The authors used cell-cycle properties from the H2B-GFP dilution data to fit lineage-tracing results by maximum likelihood parameter inference. The results show that all these datasets are in unison with the single-progenitor model (Figure 3B), with the exception of the tail inter-scale region of the skin, defending that skin and oesophageal epithelia homeostasis is equally controlled by this model of basal cell behaviour (Piedrafita et al., 2020).

Besides intrinsic ability for division, the factors that drive basal cells to make the decision to proliferate or differentiate were not yet disclosed. For instance, upon skin wounding different SC populations were shown to contribute to different compartments and change their behaviour in order to increase proliferation over differentiation until complete wound closure, only then reverting to homeostasis (Jaks et al., 2008; Lim et al., 2013; Roshan et al., 2016; Donati et al., 2017). This highlights their plasticity when challenged. More recently the concepts of local fate coordination and epidermal cell competition were brought into discussion as key players of epithelial cell dynamics (Lei and Chuong, 2018; Mesa et al., 2018; Murai et al., 2018; Piedrafita et al., 2020). SC self-renewal was shown to be driven by differentiation of neighbouring cells, supporting the concept of local fate coordination, needed to achieve a precise balance of SC activity (Mesa et al., 2018). Upon differentiation, the space left is occupied by one of the directly neighbouring progenitors which competes with the others for filling the space. Cell competition is the process of elimination of less fit cells that regulates tissue homeostasis and defence against mutant populations which ultimately could evolve to tumours (Murai et al., 2018). Cell competition has been found in different tissues, such as skin, oral mucosa, intestine and oesophagus, and it is often associated with differential gene expression between competing cells (Klein et al., 2010; Snippert et al., 2010b; Klein and Simons, 2011; Alcolea et al., 2014; Lynch et al., 2017; Martincorena et al., 2018; Corominas-Murtra et al., 2020). Clone growth is restricted by the limited size of the proliferating compartment; therefore, since the epithelial progenitors reside in a continuous sheet with no barriers, the mutant clones can expand and collide with other surrounding progenitors. When these encounter similar competitive cells, the fate of the mutant clones reverts to a homeostatic behaviour (Hall et al., 2018; Martincorena et al., 2018; Colom et al., 2020). Both the skin and oesophageal local fate coordination and competition events were shown to be compatible with the single-progenitor model, regulating epithelial cell dynamics governed by stochastic, but, also biased progenitor fates (Piedrafita et al., 2020).

The oral epithelia SCs remain largely uncharacterised and the attribution of the EPU model of homeostasis was often assumed from studies performed in other epithelia, mainly skin (Alonso and Fuchs, 2003; Dabelsteen and Mackenzie, 2006; Thomson, 2020). More recent studies have been exploring different regions of the oral cavity and pointing to which model of epithelial homeostasis suits best with the results. Some studies have defended the EPU model for mouse tongue SC patterns (Luo et al., 2009; Tanaka et al., 2013; Tang et al., 2013). The specialised epithelium of the tongue was demonstrated to house two different SC niches, one in the basal layer where long-term progenitors characterised as Krt14^+^Krt5^+^Trp63^+^Sox2^*Low*^ maintain the physiology of filiform and fungiform papillae, circumvallate papilla and soft palate, and the other is located outside the taste buds and is a Krt14^+^Krt5^+^Trp63^+^Sox2^+^ population of bipotential progenitor cells which give rise to both taste pore keratinocytes and receptor cells of the taste buds (Okubo et al., 2009). Jones and colleagues presented a study using lineage-tracing, label retention and single-cell RNA-sequencing that argues against the EPU model, stating that both the dorsal tongue and buccal mucosa epithelia are maintained by the single-progenitor model of homeostasis (Figure 3B). Additionally, the oral epithelial progenitor cells responded to epithelial damage by amending their daughter cell fates (Jones et al., 2019). Label-retaining cells were not found in tongue and oropharynx epithelia, however they observed that the hard palate displays a heterogeneous pattern of proliferation. The palate rugae junctional zone was proposed to hold a reserve SC niche, as these present characteristics of quiescence, self-renewal by symmetric cell divisions, Lrig1 expression, and activation after injury (Byrd et al., 2019). Furthermore, Lrig1 plays a critical role in regulating the oral epithelial SCs of the hard palate: upon decrease of Lrig1 expression, cells exit their quiescence mode, inducing proliferation in response to stress and injury (Byrd et al., 2019). Another study points to a *Wn*t-responsive long-lived SC population in the hard and soft palates basally located, responsible for homeostasis and response to injury. However, the soft palate showed a more robust and faster re-epithelialisation (Yuan et al., 2019).

Overall, the oral cavity is composed of a variety of types of epithelia with different lineage origins (Rothova et al., 2012) that serve distinct functions. More studies are needed to unveil the mechanisms underlying normal physiology of these tissues.

The main differences between epithelia of the skin, oesophagus and oral cavity, are their function, external microenvironment and differentiation markers. Their SCs are also estimated to divide at different rates: proliferating cells on the oesophagus and the oral mucosa divide on average every 2.4 days, while on the epidermis on average between 3.5 and 6 days, depending on the body region (Jones et al., 2019; Piedrafita et al., 2020). Furthermore, tissue expansion during growth or in adulthood (for example, ventral skin during pregnancy) also regulates SC division rate and global behaviour. This further supports the notion that SC behaviour is regulated by a combination of molecular and mechanical cues that regulate tissue microenvironment and cell behaviour (Vining and Mooney, 2017; Li et al., 2018; Shyer et al., 2018; Aragona et al., 2020; Biggs et al., 2020; Mcginn et al., 2021). Importantly, one of the crucial components of the microenvironment surrounding epithelial progenitor cells are fibroblasts. It has been shown that different fibroblast subpopulations which carry regionally intrinsic signals, determine the behaviour of adult epithelial cells, namely in skin and oral mucosa (Locke et al., 2008; Rinkevich et al., 2015; Yang et al., 2017; Abbasi et al., 2020). For instance, in the gingiva structure, the gingival and the junctional epithelia are phenotypically distinct. This is in part due to heterogeneous resident fibroblasts that provide different support to the epithelial growth and differentiation (Locke et al., 2008). These interactions between epithelial and subepithelial tissues hold a key role in tissue repair (McKeown et al., 2003).

## Wound Repair Mechanisms in Skin, Oesophagus, and Oral Epithelia

### Skin Wound Healing Process

Mammalian epithelia are prepared to respond to assaults to the normal tissue homeostasis, including physical, chemical and biological stress that often result in wounding. Skin wound healing response has been extensively studied giving cues to what may also be happening in the process of wound healing in other tissues.

Wound healing response begins right after injury and comprises a series of coordinated events that make part of a highly dynamic process. Although there are variations among different species, the mammalian wound healing follows a general pattern organised in four main phases: haemostasis, inflammation, proliferation and remodelling. As a very tightly regulated mechanism, minor changes could lead to impaired healing (Gurtner et al., 2008; Shaw and Martin, 2009).

The first phase, haemostasis, is triggered by damaged blood vessels leading to bleeding. At first, blood vessels constrict to stop blood flow, platelets are activated and aggregate in order to seal the ruptured blood vessel wall. Consequently, a fibrin clot is formed to keep the platelets and blood cells in the wound site. The clot holds a role as an initial matrix scaffold rich in growth factors that will recruit cells for further wound healing stages (Etulain, 2018). Platelets were also shown to produce a positive effect on mouse skin wound healing by enhancing the angiogenic potential of mesenchymal SCs (Levoux et al., 2021). Upon activation, platelets release respiration-competent mitochondria that are internalised by recipient mesenchymal SCs, where it stimulates their metabolism to produce increased levels of certain metabolites. Particularly citrate, which works as the main fuel for *de novo* fatty acid synthesis that in turn increase secretion of pro-angiogenic factors by mesenchymal SCs (Levoux et al., 2021). The inflammatory phase of wounding response starts with the recruitment of immune cells that travel to the injury site in order to remove pathogenic microbes. Following the platelets, neutrophils and monocytes, which differentiate into macrophages, are recruited. These have been shown to also participate in later phases of wound healing, contributing largely to cytokines and growth factors secretion, which activates and recruits other cells important for the wound healing process (Park and Barbul, 2004). The proliferation phase of wound healing comprises the rebuild of the wound site where new tissue is generated. In skin, it starts from 2 to 10 days after injury and can last for up to 3 weeks. This phase is characterised by abundant formation of a highly vascularised granulation tissue through deposition of ECM by fibroblasts (mainly composed of type III collagen), replacing the fibrin matrix (Rognoni et al., 2018). Keratinocytes and endothelial cells are recruited and activated in the wound site, actively promoting re-epithelialisation and neovascularization. Fibroblasts in the wound bed will transition to an activated state, myofibroblasts, which will not only contribute for ECM deposition but also to allow wound closure through contraction (Hinz, 2007; Velnar et al., 2009; Darby et al., 2014; Rognoni et al., 2018; DesJardins-Park et al., 2019). Importantly, in mice the presence of a thin muscular layer, the *panniculus carnosus*, promotes skin contraction and union of the wound edges; while human skin is devoid of this muscular layer (Zomer and Trentin, 2018).

The last and longest phase of wound healing is the remodelling phase which starts around week 3 and can last for up to more than 1 year. During this phase the type III collagen is actively remodelled to type I collagen by fibroblasts, macrophages and endothelial cells, which secrete MMPs (Martins et al., 2013). This rearrangement of collagen fibres allows the new skin area to become stronger and reduces scar thickness over time; however, the tensile strength of the wound area can only reach 80% compared to unwounded tissue (Gurtner et al., 2008; Xue and Jackson, 2015; Marshall et al., 2018; DesJardins-Park et al., 2019). Recent findings highlighted the role of two fibroblast-expressing transcription factors in wound healing impairment and scarring of the skin: the cyclin-dependent kinase inhibitor p21 and the gap junction alpha-1 protein Connexin43 (Jiang et al., 2020b; Wan et al., 2021).

Wound healing in the oral cavity has a different timeline from the skin. Epithelial cells start migrating and proliferating 24h post wounding and, for wound areas up to 5mm, a complete re-epithelialisation is reached by day 2 to 3 in oral mucosa, while in skin it would take up to 7 days (Szpaderska et al., 2003; Chen et al., 2010; Larjava, 2012; Glim et al., 2013; Iglesias-Bartolome et al., 2018). Inflammation peaks at days 2 to 3 as well and is resolved by day 6 (Bodner et al., 1993; Szpaderska et al., 2003; Iglesias-Bartolome et al., 2018). The further proliferation phase takes place very early from day 2 to 7, being followed by the remodelling of collagen (Bodner et al., 1993; Nikoloudaki et al., 2020).

The cellular and molecular mechanisms underlying oesophageal wound healing have recently attracted attention. Despite comparisons with gastric healing, the similarities to the epidermis have also prompted studies to disclose possible critical players in oesophageal response to wounding (Baatar et al., 2002a, b; Chai et al., 2007; Tarnawski and Ahluwalia, 2012; Jönsson et al., 2016; Tabola et al., 2016; Cai et al., 2018; Komaki et al., 2019; Boudaka et al., 2020).

### The Multifaceted Outcomes of Scarring

The regeneration of a skin wound will lead to fine scar formation in superficial injuries. However, there are more complex outcomes for scarring including widespread scars, atrophic scars, scar contractures, hypertrophic scars, and keloid scars (Karppinen et al., 2019). Hypertrophic and keloid scars are pathological outcomes that come with devastating consequences for patients, such as pain and itching. Hypertrophic scars are lifted, erythematous, pruritic lesions confined to the wound boundaries while keloids are benign fibroproliferative dermal scars, growing beyond the wound margins (Bayat et al., 2003; Brown et al., 2008; Karppinen et al., 2019). Given their quasi-neoplastic tendencies, it has been argued that keloids should be classified as a pathological disease rather than a scar (Ud-Din and Bayat, 2020). Besides minor traumatic wounds and acne, other cases can arise from clinical surgeries, chemical and thermal burns or in consequence of allergic reactions. Self-harm scarring and combat wounds also a matter of concern (Mitchell et al., 2019; Johnson et al., 2020). The traumatic wounds in the hostility of war context come with exposure of bone, ligaments and tendons, as well as contamination, and the limited available resources in conflict zones’ hospitals impede the treatment of these wounds (Johnson et al., 2020).

On the one hand, skin scars carry long-term psychosocial effects, including anxiety and avoiding social interaction. This behaviour will interfere with future work life and relationships. In some contexts, scars result from traumatising events and bury a psychological meaning (Brown et al., 2008; Gibson et al., 2018; Mitchell et al., 2019). On the other hand, while visible skin scarring implies a social burden, oral and oesophagus scarring result in difficulties swallowing food and weight loss (Campos et al., 2020).

The fibrous tissues formed upon oesophageal injury are named oesophageal strictures and are mainly a consequence of various benign and malignant disorders. Some other causes include radiation therapy and caustic ingestions. Peptic strictures are caused by gastroesophageal reflux disease when stomach acid damages the oesophagus epithelium over time (Yamasaki et al., 2016). Stricture formation may result from extended endoscopic mucosal resection and submucosal dissection, two techniques used for treatment of superficial gastrointestinal neoplasia, gastric cancer and superficial Barrett’s oesophagus (Yang et al., 2019; Huang et al., 2020). The oesophageal stricture may be persistent or recurrent despite application of several therapies. These can cause complications such as solid and liquid dysphagia, regurgitations or aspiration, abdominal and chest pain as well as obstruction of the oesophagus (Ferguson, 2005).

Compared to the skin and oesophagus, the oral mucosa has an exceptional regenerative ability, being much less prone to scar formation. Despite owning this scar-free healing capacity, there are some particular cases of scar formation. The mucosal trauma applied by oral and perioral piercings may in some rare cases cause complications. Moreover, the oral mucosa may form a keloid or hypertrophic scar as a consequence of medication or of systematic disease (Escudero-Castaño et al., 2008). Additional scar formation may be a consequence of the cleft lip, palate and gum reconstruction, as well as removal of benign and malignant oral tumours (Goodacre and Swan, 2008; Chang et al., 2012; Fierz et al., 2013; Botticelli et al., 2019). Some diseases are also associated with oral mucosal fibrosis, including submucous fibrosis, pemphigus vulgaris and cicatricial pemphigoid, lichen planus, epidermolysis bullosa and proliferative verrucous leukoplakia (Evans, 2017). These can lead to failure in normal growth and restricted oral aperture (Wright, 2010). The molecular mechanisms underpinning these changes in oral wound healing are a subject of ongoing research.

### The Outstanding Regenerative Potential of Oral Mucosa – Scarless Wound Healing

**FIGURE 4:**
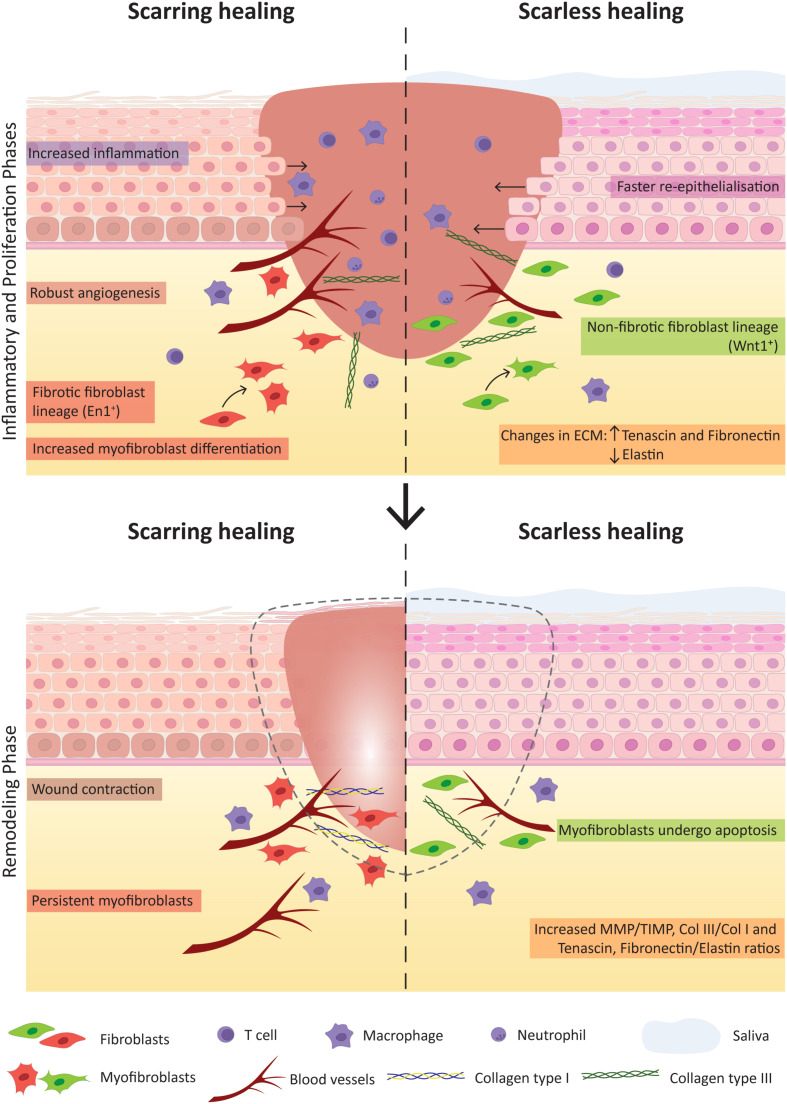
Key factors contributing for scarring and scarless wound healing. The comparison of the inflammatory and proliferation phases **(Top)** and the remodelling phase **(Bottom)** of wound healing highlight crucial factors that notably contribute to the distinct healing outcome of skin (with scar formation) and oral mucosa (scarless).

The only adult tissue with the potential to heal with minimal scar formation is the oral mucosa. This capacity is comparable to foetal skin scarless healing, occurring during the first and second trimesters of pregnancy (Rowlatt, 1979; Colwell et al., 2005; Karppinen et al., 2019). Several studies have evidenced that oral mucosa heals faster than skin (Szpaderska et al., 2003; Mak et al., 2009; Chen et al., 2010; Iglesias-Bartolome et al., 2018). Studies exploring the mechanisms of oral repair have allowed to point key differences responsible for the superior outcome when compared to skin (Figure 4). The main differences are:

(1) Environment: the oral mucosa comes in contact with a very different environment compared to skin. The external factors such as saliva and the oral microbiota have been shown to play a role in oral wound healing (Hutson et al., 1979; Bodner et al., 1993; Su et al., 2018). The oral microbiota was shown to affect wound repair through secretion of lipopolysaccharides which maintains oral mesenchymal SCs homeostasis via miRNA-21/Sp1/telomerase reverse transcriptase pathway (Su et al., 2018). Bacteria may accelerate wound healing with beneficial effects in the immune response, granulation tissue and collagen formation (Jones et al., 2004). The positive role of saliva in wound repair has been explained by it being composed of growth factors such as the epidermal growth factor and peptides as histatins with antimicrobial function, responsible for enhanced oral keratinocyte and fibroblast migration. Therefore, saliva modulates oral and eventually skin wound healing mediating the inflammatory response (Figure 4; Zelles et al., 1995; Oudhoff et al., 2008; Boink et al., 2016; Neves et al., 2019).
(2) Inflammation: the inflammatory response in oral wounds was shown to be reduced and to be concluded earlier than in skin wounds (Mak et al., 2009). In fact, there is much evidence linking excessive fibrosis with a strong inflammatory response to injury (Shaw et al., 2010; Wang et al., 2015). The number of immune cells such as neutrophils, macrophages and T cells in oral wound response is reduced when compared to skin, and linked with reduced levels of inflammatory cytokines [as interleukin (IL)-23, IL-24, IL-6, IL-8, tumour necrosis factor alpha (TNF-α)] and pro-fibrotic cytokines [transforming growth factor β1 (TGF-β1)], leading to decreased recruitment of inflammatory cells, and elevated anti-fibrotic cytokine TGF-β3 (Szpaderska et al., 2003; Schrementi et al., 2008; Chen et al., 2010; Glim et al., 2013). The reduced inflammation observed in the oral tissues during wound healing is a reflection of a tissue with the right tools to respond more efficiently. The local oral defences are constantly stimulated by the commensal microbiota and mastication, which trigger cellular crosstalk essential for homeostasis maintenance (Moutsopoulos and Konkel, 2018; Caetano et al., 2021; Williams et al., 2021). Another key immunosuppressive population in the mouth is Foxp3^+^ regulatory T cells (Park et al., 2018). Inflammatory response in the oral mucosa can be significantly amplified in cases of chemotherapy treatment or as a consequence of systemic conditions involving autoimmune responses, as of lichen planus, leading to increased probability of fibrotic tissue formation (Roopashree et al., 2010; Park et al., 2018; Basile et al., 2019). In fact the local oral tissue immunity can affect and be affected by extra-oral diseases (Moutsopoulos and Konkel, 2018; Kitamoto et al., 2020).
(3) Angiogenesis: reduced angiogenesis could be expected to impair healing, though some studies have proven that inhibition of the angiogenic response in oral wounds is linked to reduced scar formation (Szpaderska et al., 2003; Wilgus et al., 2008). Angiogenesis can directly affect scar formation through oedema, apoptosis and transition of recruited pericytes to an activated fibroblast phenotype (Dulmovits and Herman, 2012; Johnson and Di Pietro, 2013; DiPietro, 2016). Angiogenesis and the inflammatory response act together as inflammatory cells release pro-angiogenic molecules (vascular endothelial growth factor (VEGF) and CXC chemokines) to promote capillary growth, which in turn will support the inflammatory response (Lucas et al., 2010; DiPietro, 2016).
(4) Keratinocyte proliferation: the oral epithelia present faster re-epithelialisation (Szpaderska et al., 2003; Chen et al., 2010; Glim et al., 2014). Oral keratinocytes present higher proliferative potential and are less differentiated than skin keratinocytes therefore contributing with a greater regenerative potential (Glim et al., 2014; Turabelidze et al., 2014; Iglesias-Bartolome et al., 2018).
(5) Fibroblasts: the major players of the proliferative phase of wound healing are fibroblasts that are responsible for collagen deposition and wound contraction, being critical players in the process of scarring. Several studies have investigated how different fibroblast lineages contribute to oral and to skin wound healing (Rinkevich et al., 2015; Gölz et al., 2016; Jiang et al., 2020a). Apart from the *Engrailed1*-lineage-positive fibroblast subpopulation, the study from Rinkevich and colleagues reports a *Wnt1*-lineage-positive population in the oral dermis tightly linked to the non-fibrotic healing that characterises the oral mucosa. A reciprocal transplantation of these oral mucosal- and skin-derived fibroblast populations performed in mice revealed that these mimic the response of the tissue of origin. Thus, the grafting of *Wnt1*-lineage-positive oral fibroblasts in skin resulted in decreased scar tissue formation while skin fibroblasts contributed for a scar-like tissue formation in the oral wound site, proving that the oral fibroblast lineage is determinant for the scarless healing of the oral mucosa (Rinkevich et al., 2015). Comparison between dermal and gingival fibroblasts showed that the latter have increased *in vitro* proliferation, migration and efficiency in remodelling connective tissue (Chaussain et al., 2002; Boink et al., 2016; Isaac et al., 2018), however contradictory results were reported in regard to the contraction capacity of oral fibroblasts (Lygoe et al., 2007; Mak et al., 2009). Recent studies in mice revealed the contribution of subcutaneous fascia fibroblasts to large deep skin wound healing through deposition of matrix and further contraction into a more exuberant scar matrix architecture (Correa-Gallegos et al., 2019). This is mediated by migration and swarming to the surface involving N-cadherin-mediated cell-cell adhesion. Further experiments with an *ex vivo* explant technique termed scar-like tissue in a dish (SCAD) using oral mucosa (without fascia) showed that swarming is absent and N-cadherin is minimally expressed, agreeing with its typical scarless healing phenotype (Jiang et al., 2020a).
  Several studies explored the differential response of oral and dermal fibroblasts to TGFβ1, a cytokine known to mediate fibroblast to myofibroblast differentiation and up-regulating the α-smooth muscle actin (αSMA) in these cells. Oral fibroblasts were shown not only to express higher basal levels of αSMA but also have higher number of αSMA-positive myofibroblasts in oral mucosal wounds (Lygoe et al., 2007; Mak et al., 2009). However, these were shown to resist more to TGFβ1-controlled myofibroblast differentiation, together with decreased levels of TGFβ1 in oral wounds, and supporting its non-scarring phenotype (Meran et al., 2007). This can be regulated by the increased expression of the hepatocyte growth factor (Dally et al., 2017).
(6) ECM: compared to cutaneous wounds, the ECM composition of oral wounds diverges and is a key determinant for the scarless phenotype. Oral wounds showed increased expression of hyaluronic acid, tenascin and fibronectin and decreased expression of elastin (Glim et al., 2013, 2014. MMP mediate ECM remodelling and are regulated by MMP tissue inhibitors. The balance between these two molecules was shown to be important for the final healing outcome. In oral wounds the ratio between MMP and MMP tissue inhibitors is high, namely the levels of MMP 2 and 3 (Stephens et al., 2001; Glim et al., 2013). Also, the collagen III to collagen I ratio is increased in oral wounds (Glim et al., 2013; Figure 4). The pro-fibrotic matricellular protein periostin was recently shown to be involved in ECM synthesis regulation in gingival wound healing, while in skin it appears as a mediator of myofibroblast differentiation through β1 integrin-focal adhesion kinase (FAK) signalling (Nikoloudaki et al., 2020). Another study related the activation of autophagic pathways with an increase in myofibroblast differentiation and noted heterogeneity within the oral cavity, namely between buccal mucosa and gingiva. The gingival tissue showed no autophagic process upon wound repair therefore leading to less myofibroblast differentiation when comparing to buccal mucosal tissue (Vescarelli et al., 2017). It would be interesting to deepen our knowledge on the different wound healing responses associated with different tissues of the oral cavity. Overall, the surrounding environment is capable of eliciting various responses that contribute for the scarless potential of oral mucosa, nevertheless, also inside the cells molecular differences can be pointed between skin and oral mucosa.
(7) Molecular cues: transcriptomic analysis have uncovered the molecular differences between skin and oral mucosal wound healing (Chen et al., 2010; Turabelidze et al., 2014; Iglesias-Bartolome et al., 2018). Healthy oral mucosa is primed with transcriptional networks readily prepared to respond to wounding, suggesting that the oral epithelia is equipped with a specially prepared intrinsic genetic response, particularly for cellular growth and proliferation and inflammatory response (Turabelidze et al., 2014; Iglesias-Bartolome et al., 2018). Importantly, the discovery of key players in transcriptional networks directly working for a scarless healing is of major importance. For instance, the Sox2 and Pitx1 transcription factors were shown to be the master regulators of the oral mucosal wound healing response (Iglesias-Bartolome et al., 2018). However, the intrinsic features playing to scarless healing are not restricted to the protein coding genes; microRNAs were differentially expressed between skin and oral wound healing, highlighting that genetic and epigenetic response of oral mucosa through growth factor production, SC levels and cellular proliferation capacity gives this epithelium its superior final repair (Simões et al., 2019).

To conclude, the ability of the oral mucosa to heal without scarring cannot be attributed to a single feature but to key extrinsic and intrinsic factors present in all stages of the wound healing process, which are crucial to the final improved outcome.

## Exporting the Properties of Oral Epithelia – the Source for Future Therapies in Wound Repair?

Improving wound healing in skin is an unmet need. Chronic skin wounds have devastating consequences for patients and treating chronic wounds costs the UK National Health Service £5 billion per annum (Guest et al., 2015). Development of more efficient wound treatments is urgently needed to increase the quality of life of patients and to effectively reduce healthcare costs.

Reconstruction of skin or oral mucosal tissues using tissue-engineering methods resembles wound healing processes. It requires active SCs, epithelial proliferation, epithelial and fibroblast cell migration and ECM production, all processes coordinated to regenerate the new 3D tissue with similar properties and functions.

**FIGURE 5:**
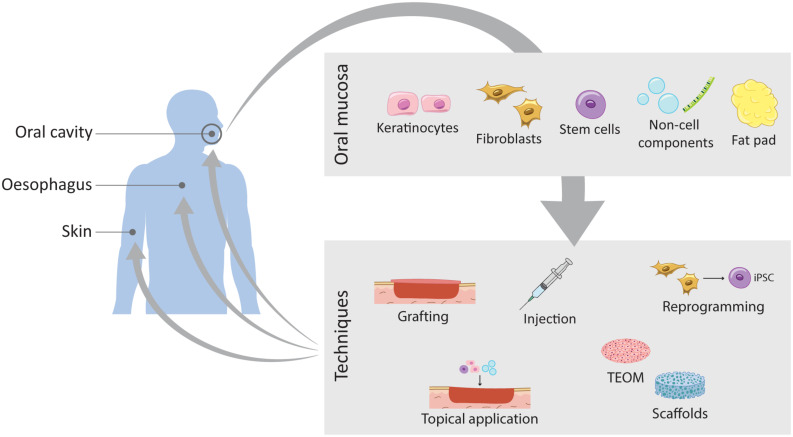
Schematic representation of the current work on wound healing improvement using oral mucosa. The oral mucosa represents a valuable source of different components that translates into different routes of exploration and expansion of its unique healing potential. Through different techniques these components can be applied to different tissues such as the oral mucosa itself, the skin and the oesophagus.

A large number of studies have been exploring SC therapies to improve skin regeneration. A major breakthrough recently published has used autologous transgenic skin epithelial cultures to regenerate an entire, fully functional epidermis from a patient with an epidermolysis bullosa disease caused by a mutation in *laminin 332* usually expressed in skin’s basement membrane (Hirsch et al., 2017). Using retrovirus bearing healthy copies of the needed gene, *LAMB3*, epithelial cells from the patient were corrected, expanded in culture and grafted back to the patient. By combining cell and gene therapy, this clinical study demonstrated a life-saving regeneration of virtually the entire epidermis. This study inspires the use of other tissues for skin regeneration. Oral mucosal cells present advantages over skin cells in therapeutic applications due to their unique scarless properties and are an easy source to harvest reducing time for surgical procedures and accelerating patient’s recovery time (Izumi et al., 2015; Chapple, 2020). However, the direct use of mucosal grafts comes with various disadvantages associated with availability of sufficient amount of donor tissue as well as other graft-associated problems, such as donor site morbidity, recipient site, pain and risk of infection (Llames et al., 2014). To overcome these problems, the clinical use of tissue-engineered oral mucosa (TEOM) is the most adopted method (Figure 5).

TEOMs are based on a scaffold matrix that provides structural support for the cells to seed, or as a scaffold used to deliver drugs or growth factors directly into the injured tissue, upon transplantation. The key factors are the optimal choice of the scaffold and the cells to seed. Collagen scaffolds are the golden standard, but advances in tissue engineering are proposing other synthetic scaffolds such as biodegradable hydrogels, as well as decellularised dermis (Figure 5). TEOM is a potential technique to reconstruct the oral cavity after tumour excision or after injury, and to repair congenital defects, such as cleft palate. Furthermore, it is a great model for *in vitro* testing of oral care products efficiency and safety, for evaluating cigarette smoke effects and to analyse cellular and molecular mechanisms of infection in the oral cavity (Chen et al., 2020; Wang et al., 2020; Zhong et al., 2020; Huang et al., 2021).

The TEOM explores the outstanding regenerative potential of the oral mucosal to reconstruct the oral cavity itself or in other tissues of the body. The following subchapters cover pre-clinical and clinical studies on the use of the oral mucosal tissue to improve the healing outcome of other intra- and extra-oral tissues (Tables 1, 2).

**TABLE 1:** Clinical application of cellular therapy using human oral mucosa cells to regenerate oral tissues or other recipient tissues.

Intra-oral					

Cell type	Method	Donor tissue	Recipient tissue	Outcome	Reference(s)
Keratinocytes 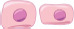	TEOM 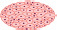	Hard palate	Tongue (intra-oral wound)	Improved tissue adhesion, speech and tongue mobility	Lauer and Schimming, 2001
		Keratinised oral mucosa on human dermis (AlloDerm^®^)	Tongue, alveolar gingiva, buccal mucosa, floor of mouth and Oropharyngeal mucosa	No postoperative pain, excellent adhesiveness and good epithelial coverage	Izumi et al., 2003
		Gingiva keratinocytes on human dermis (AlloDerm^®^)	Tongue, gingiva, buccal mucosa and alveolar ridge	Faster healing, negligible scar contracture	Hotta et al., 2007
		Hard palate keratinocytes on human dermis (AlloDerm^®^)	Gingiva	Good adhesiveness, increased gingival tissue	Izumi et al., 2013

Keratinocytes and fibroblasts 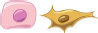	TEOM 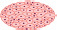	Buccal mucosa	Tongue	Good mobility of tongue, satisfactory speech, residual fibrosis	Llames et al., 2014
		Palatal mucosa	Fibula flaps for maxillary and mandibular reconstruction	Granulation tissue formation in one patient, good restoring outcome	Gil et al., 2015

Fat pad 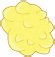	Grafting 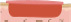	Buccal fat	Posterior alveolus and hard palate	Full recovery	Egyedi, 1977
		Buccal fat	Mid-palatal and posterior palatal fistulas	Full recovery	Ashtiani et al., 2011
		Buccal fat	Palatal fistulas	Full recovery	Yaguchi et al., 2021

Fibroblasts 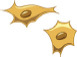	Injection 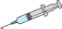	Gingiva	Gingiva	Test treatment improved papillary tissue augmentation	McGuire and Scheyer, 2007
	Scaffold 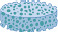	Gingiva	Gingiva	Increased gingival width, keratinised epithelium supported by dense connective tissue	Mohammadi et al., 2011
		Gingiva	Gingiva	Efficient gingival augmentation	Dominiak et al., 2012

**Extra-oral**					

**Epithelial flap**	TEOM 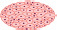	Buccal mucosa	Trachea	Faster healing, buccal mucosa and fascia form an optimised tissue combination	Delaere et al., 2001

Keratinocytes 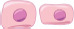	TEOM 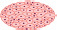	Buccal mucosa	Eye	Vision restored, no complications	Nishida et al., 2004
		Lip	Skin (scalp)	30% success of engraftment due to local infection	Iida et al., 2005
		Buccal mucosa	Oesophagus	Effective re-epithelialisation, no dysphagia or stricture formation	Ohki et al., 2012
		Buccal mucosa	Oesophagus	Safe, reduced risk for post-ESD stricture formation	Jonas et al., 2016
		Buccal mucosa	Oesophagus	Short post-ESD ulcer healing period, successful cell sheet fabrication, transport and transplantation.	Yamaguchi et al., 2017

Lingual tissue	Grafting 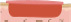	Ventrolateral tongue	Urethra	Good success rates of reconstruction of short strictures, combination with buccal mucosa for longer grafts	Simonato et al., 2008

Buccal mucosal cells	TEOM 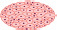	Buccal mucosa	Urethra	Safe and effective anterior urethroplasty	Barbagli et al., 2018

*Comparison of the outcomes according to the tissue of origin, the therapeutic method used and the recipient tissue. TEOM, tissue-engineered oral mucosa; ESD, endoscopic submucosal dissection.*

**TABLE 2:** Pre-clinical studies with oral mucosa.

*In vivo*						

Species	Cell type or component	Method	Donor tissue	Recipient tissue	Outcome	Reference(s)
**Mouse**	SCs	Injection	Deciduous teeth	Skin	Accelerated wound healing	Nishino et al., 2011
	Keratinocytes	Topical application	Human gingiva	Skin	Rapid re-epithelialisation	Kim et al., 2013
	Fibroblasts	Injection	Buccal mucosa	Skin	Reduced scarring, lineage-dependent behaviour	Rinkevich et al., 2015
	SC/progenitor cells	Salisphere cell transplantation	Human submandibular salivary gland	Mouse submandibular salivary gland	Rescue of saliva production	Pringle et al., 2016
	Keratinocytes and fibroblasts	TEOM	Oral mucosa (non-specified)	Skin	Faster wound healing, reduced scarring	Roh et al., 2017
	miRNA-31 mimic	Injection	Hard palate	Skin	Significant acceleration of wound closure	Chen et al., 2019
	Keratinocytes and fibroblasts	TEOM	Human oral mucosa	Skin	Accelerated wound healing, reduced scarring	Lee et al., 2019
	Exosomes	Injection	Human saliva	Skin	Efficient wound healing through promotion of angiogenesis	Mi et al., 2020
	SCs	Injection	Oral mucosa (non-specified)	Skin	Accelerated wound healing	Kuperman et al., 2020

**Rat**	Keratinocytes	TEOM	Oral mucosa (non-specified)	Uterus	Highly effective against intrauterine adhesions	Kuramoto et al., 2015
	Keratinocytes and fibroblasts	pre-vascularized TEOM	Oral mucosa (non-specified)	Buccal mucosa	Accelerated and more efficient healing	Lee et al., 2017
	Exosomes	Hydrogel topical application	Human gingival mesenchymal SCs	Skin	Promotion of re-epithelialisation, deposition and remodelling of ECM	Shi et al., 2017
	Keratinocytes and fibroblasts	TEOM	Buccal mucosa	Skin	Accelerated wound healing, reduced scarring	Lee et al., 2018
	Dental pulp SCs	Injection via tail vein	Upper and lower incisors	Oesophagus	Improved healing	Zhang et al., 2018
	EGF, HA, bFGF and lysozyme	Biomimetic hydrogel	Commercial	Skin	Accelerated wound healing, reduced scarring	Kong et al., 2019
	Mucosal tissue	Grafting	Tongue	Skin	Lower levels of EGF and VEGF-C	Qi et al., 2019
	Exosomes	Topical application	Human buccal epithelial cell sheets	Skin	Significant acceleration of wound closure	Sjöqvist et al., 2019

**Dog**	Keratinocytes	TEOM	Buccal mucosa	Oesophagus	Complete faster wound healing, no stenosis	Ohki et al., 2006
	Keratinocytes and fibroblasts	TEOM	Oral mucosa (non-specified)	Oesophagus	Good distensibility and epithelial thickness, successful oesophageal replacement	Nakase et al., 2008
	Keratinocytes	TEOM	Buccal mucosa	Oesophagus	Successful attachment and re-epithelisation	Takagi et al., 2010

**Rabbit**	Dental pulp SCs	TEOM	Human deciduous teeth	Eye	Corneal reconstruction	Gomes et al., 2010
	Keratinocytes	TEOM	Buccal mucosa	Urethra	Urethroplasty reconstruction	Yudintceva et al., 2020

**Goat**	Epithelial graft	Grafting	Oral mucosa (non-specified)	Trachea	Coverage of the constructed trachea lumen	Li et al., 2019

**Pig**	Keratinocytes	Injection	Buccal mucosa	Oesophagus	Improved re-epithelisation, reduced risk of stenosis and contraction	Sakurai et al., 2007

***In vitro***						

**Species**	**Cell type**	**Method**	**Donor tissue**	**Outcome**		**Reference(s)**

**Human**	Keratinocytes	TEOM	Gingiva	Fabrication of oral mucosal equivalent similar to the native tissue		Yoshizawa et al., 2004
	Fibroblasts	Reprogramming	Buccal mucosa	Efficient reprogramming into induced pluripotent SCs		Miyoshi et al., 2010
	Keratinocytes	TEOM	Cryopreserved lip mucosa	Successful fabrication of oral mucosa equivalents		Xiong et al., 2010
	Fibroblasts	Low-level laser therapy	Cell line	Increased cell number and migration		Basso et al., 2012
	Keratinocytes	TEOM	Lip	Fabrication of 3D human lip skin equivalent		Peramo et al., 2012
	Keratinocytes	TEOM	Keratinised oral mucosa	Development of large TEOM		Kato et al., 2015
	Fibroblasts and immortalised OKF6/TERET-2 oral keratinocytes	TEOM	Gingiva	Development of 3D bone-oral mucosa model		Almela et al., 2016
	Fibroblasts	Feeder cells	Gingiva	Improved cell proliferation, promising candidate feeder cells		Yu et al., 2016
	Fibroblasts	*In vitro* differentiation, feeder cells	Oral mucosa (non-specified)	Fabrication of corneal epithelial sheets, multipotent differentiation into mesenchymal or neural crest-derived cells, good source of feeder cells		Higa et al., 2017
	Keratinocytes and fibroblasts	Scaffolds	Buccal mucosa	Tri-layer micro-nano-3D porous synthetic scaffold mimics normal human oral mucosa, minimal contraction, good mechanical properties		Simsek et al., 2018
	Keratinocytes and fibroblasts	TEOM	Gingiva	Development of 3D epithelium and lamina propria		Nishiyama et al., 2019

**Pig**	Keratinocytes	TEOM	Buccal mucosa	Culture on acellular scaffolds		Poghosyan et al., 2013

**Dog**	Keratinocytes	TEOM	Buccal mucosa	Successful construction of TEOM with adipose derived SCs and small intestine submucosa		Zhang et al., 2021

*Comparison of the outcomes according to the animal species, the cell type or non-cell component, the method used and the recipient tissue. *TEOM, tissue-engineered oral mucosa; EGF, epidermal growth factor; HA, hyaluronic acid; bFGF, basic fibroblast growth factor; VEGF-C, vascular endothelial growth factor C.**

### Exploring the Use of Oral Mucosa for Oral Tissue Repair

The human clinical application of oral mucosal scarless potential and exceptional properties for repair is still scarce, however the number of case reports and pilot studies has been growing (Figure 5 and Table 1). TEOM produced *ex vivo* from autologous keratinocytes from the hard palate or gingiva were successfully used for reconstruction of intra-oral lining tissues and periodontal plastic surgeries (Lauer and Schimming, 2001; Izumi et al., 2003; Hotta et al., 2007), while full-thickness TEOM combined with fibula flap allowed for the lining reconstruction of maxilla and mandible (Gil et al., 2015). Other cases of congenital anomalies such as hemifacial microsomia, ankyloglossia (tongue-tie) and cleft palate were treated with TEOM yielding satisfactory outcomes (Llames et al., 2014; Hixon et al., 2019). The use of TEOM to repair mucogingival defects demonstrated its capacity to integrate and vascularise (Izumi et al., 2013), however this technique still needs to be improved to avoid postoperative wound shrinkage.

The buccal fat pad flap is reported to be a reliable and effective flap with clinical application in reconstruction of oral defects due to its high vascularity, reducing tissue hypoxia and improving graft survival. This has been used to treat oroantral fistula, congenital defects such as the cleft palate, osteonecrosis of the jawbone and defects induced by removal of tumours or cysts (Egyedi, 1977; Ashtiani et al., 2011; Kim et al., 2017; Yaguchi et al., 2021).

The clinical use of oral-derived SCs is still limited. Oral SCs have been derived from dental pulp, periodontal ligament, exfoliated deciduous teeth, apical papilla, dental follicle, gingiva, oral mucosa, salivary glands and alveolar bone (Kanwal et al., 2017; Bryja et al., 2019; Sanz et al., 2019). The work with oral SCs for hard and soft tissue regeneration within the oral cavity has focused on the use of oral SCs for reconstructing periodontal, bone, dentin and pulp tissues (Seo et al., 2004; Feng et al., 2010; Giuliani et al., 2013; Shiehzadeh et al., 2014; Surendran and Sivamurthy, 2015; Chen et al., 2016; Kanwal et al., 2017). Human gingival and mouse palatal epithelial cells were used to develop teeth in combination with mouse embryonic tooth mesenchyme following transplantation into renal capsules (Nakagawa et al., 2009; Volponi et al., 2013). The combination of human oral epithelial cells and dental pulp SCs using a matrigel as scaffold allowed the 3D construction of an epithelial invagination model, an important feature of early tooth development (Xiao and Tsutsui, 2012). Furthermore, human salivary gland-derived SCs were used to restore saliva production after radiation of salivary glands, opening doors for the treatment of hyposalivation resulting from head and neck cancer radiotherapy (Pringle et al., 2016).

Several clinical studies explored the potential of using oral mucosal fibroblasts for gingival tissue augmentation. Autologous gingival fibroblasts seeded in different scaffolds improved keratinised tissue formation (Mohammadi et al., 2011; Dominiak et al., 2012). Additionally, the injection of autologous fibroblasts harvested from keratinised tissue from the maxillary tuberosity in interdental papillary recession defects improved the papillary tissue augmentation (McGuire and Scheyer, 2007; Table 1).

In addition to clinical studies, *in vitro* and *in vivo* research is progressing with more viable alternatives (Table 2). A large size *ex vivo* fabricated oral mucosal equivalent was successfully achieved using higher cell seeding density of oral keratinocytes and a thinner AlloDerm scaffold, reaching a final 15cm^2^ size, which can be applied in the reconstruction of significant soft tissue defects (Kato et al., 2015). Cryopreservation of abundant lip mucosa tissues harvested upon cleft lip repair proved to be a useful approach to biobank oral keratinocytes for TEOMs. The 4- to 6-month cryopreservation did not affect the characteristics of the TEOM when compared with the equivalents engineered from fresh lip and palate (Xiong et al., 2010). Furthermore, pre-vascularised oral mucosal cell sheets grafted into deep wounds in the buccal region of rats healed more rapidly and without fibrosis (Lee et al., 2017).

Using cell surface coating through layer-by-layer assembled ECM films succeeded in creating the 3D oral mucosal equivalents composed of epithelium, lamina propria and blood capillaries, recreating the tissue cellular heterogeneity (Nishiyama et al., 2019). Furthermore, the *in vitro* incorporation of oral mucosa and bone components in a composite scaffold model that mimics the natural structure of alveolar bone with an overlying oral mucosa was achieved and is a possible future application in cleft palate repair (Almela et al., 2016; Hixon et al., 2019).

Oral mucosal fibroblasts were recently reprogrammed into induced pluripotent SCs (Miyoshi et al., 2010) and were shown to be efficient feeders for induced pluripotent SCs expansion (Yu et al., 2016; Figure 5). This represents a promising tool for *ex vivo* cell expansion.

Recent technologies have been exploring the use of cell-free therapies for oral maxillofacial regeneration, particularly the use of extracellular vesicles (Figure 5; reviewed in Lv et al., 2019; Shi et al., 2020). This technology overcomes the shortcoming of cells and instead explores the cell paracrine effects that can activate endogenous repair pathways (Jiang et al., 2017; Zheng et al., 2018; Ren, 2019). As in TEOMs, it remains important to consider the origin and the differentiation status of the secreting cells to guarantee not only improved outcome but also safe clinical applications.

### Exploring Oral Mucosal Properties to Improve Skin and Oesophageal Repair

It is well known that adult skin wounds are frequently accompanied by scar formation that can become fibrotic, while oral mucosal wounds heal in an accelerated fashion, displaying minimal scar formation (see section “Wound Repair Mechanisms in Skin, Oesophagus, and Oral Epithelia”). While surgical reconstruction of the oral cavity with skin grafts has been easily and routinely accomplished for a long time, particularly after tumour resection, the clinical application of oral mucosa grafts into skin has been less explored (Schramm and Myers, 1980; Schramm et al., 1983; de Bree et al., 2008). However, exploring the scarless potential of the oral mucosa offers an exciting strategy to accelerate skin wound healing and to improve the quality of life for patients with chronic wounds. There have been promising studies around this topic, but few clinical studies. Iida and colleagues used TEOMs based on an acellular allogeneic dermal matrix grafted into scalp skin with extensive deep skin burn showing 30% of the graft efficacy (Iida et al., 2005; Table 1).

*In vitro* studies have demonstrated the potential of oral mucosal fibroblasts, to improve wound healing after biostimulation with low-level laser therapy (Basso et al., 2012). A recent study by Kong and colleagues engineered a biomimetic gel inspired by the characteristics of oral mucosal wound healing. The hydrogel was loaded with epidermal growth factor, basic fibroblast growth factor, lysozyme and hyaluronic acid, as these were observed to be highly expressed in the oral mucosal wound healing when comparing to skin (Kong et al., 2019). The authors were able to simulate the oral mucosal trauma microenvironment through controlled release of these molecules resorting to microspheres and chitosan thermo-sensitive gels. The use of this biomimetic hydrogel in skin wounds of rats resulted in rapid wound healing and reduced scar formation (Kong et al., 2019).

As for pre-clinical studies, topical grafting of human oral keratinocytes onto skin wounds in nude mice improved regeneration of skin wounds, with increased production of keratinocyte growth factor and cytokines IL-6, and IL-1α (Kim et al., 2013).

SCs from human exfoliated deciduous teeth and oral mucosa were shown to improve skin wound healing in mice when injected around the wound or topically applied onto the wound bed highlighting the plasticity of different SC types that could be used to regenerate skin (Nishino et al., 2011; Kuperman et al., 2020). Other studies have explored the potential of oral mucosal engineered cell sheets to apply on skin excisional and burn wounds. All reported results indicated the plasticity of the cell sheets in adapting to the skin wounds, the contribution to accelerated healing and limited scar formation (Roh et al., 2017; Lee et al., 2018, 2019).

The implementation of recent knowledge in the prevention of oesophageal stricture after endoscopic submucosal dissection has attracted increasing attention. Autologous oral mucosal keratinocytes were endoscopically implanted after oesophageal resection in a porcine model, resulting in accelerated re-epithelialisation and wound healing (Sakurai et al., 2007). On a “bench to bedside” approach, tissue-engineered autologous oral epithelial cell sheets and full-thickness substitutes were endoscopically transplanted into an oesophageal ulcer immediately after a large endoscopic submucosal dissection, showing excellent results with no cases of dysphagia or stricture formation (Ohki et al., 2006, 2012, 2015; Arakelian et al., 2018; Ohki and Yamamoto, 2020). The first clinical trial using cell sheet technology for oesophageal reconstruction in Europe used cell sheets from autologous oral epithelial cells, transplanted right after endoscopic submucosal dissection of Barrett’s neoplasms, resulting in decreased risk and extent of strictures (Jonas et al., 2016). On a canine model, the replacement of the full-circumference and full-thickness intrathoracic oesophagus was achieved using autologous oral keratinocytes and fibroblasts seeded on amniotic membrane, sheeted on polyglycolic acid filled with smooth muscle tissue (Nakase et al., 2008), a technique later also achieved without animal-derived materials that could compromise future human clinical trials (Takagi et al., 2010). On a porcine model, an *in vitro* engineered oesophageal substitute was obtained with an acellular small intestinal submucosa scaffold seeded with autologous skeletal myoblasts, covered with a human amniotic membrane and seeded with autologous oral epithelial cells (Poghosyan et al., 2013).

Other non-cellular-based approaches have also emerged which explore the benefits of oral microRNA and exosomes for skin and oesophageal wound repair (Shi et al., 2017; Chen et al., 2019; Sjöqvist et al., 2019; Mi et al., 2020).

Finally, an interesting and challenging study tested the safety and feasibility of transplanting engineered autologous oral mucosal cell sheets in patients who had undergone extensive endoscopic submucosal dissection for oesophageal squamous cell carcinoma removal. The challenge of this study was the use on non-autologous cell sheets produced 1200km away from the patients that were transported by air for 7h before transplantation (Yamaguchi et al., 2017). Such studies are of major relevance when thinking about future tissue-engineering therapies reaching remote hospitals with no tissue engineering facilities, or hospitals in conflict zones.

### Exploring the Use of Oral Mucosa for the Repair of Other Tissues

The use of oral mucosa to improve wound healing in other tissues has taken its first steps with successful applications in the clinical field, such as in urethral reconstruction and stricture repair through grafting, with recent research focused on improving the TEOM for better outcomes (Simonato et al., 2008; Barbagli and Lazzeri, 2015; Horiguchi, 2017; Barbagli et al., 2018; Simsek et al., 2018; Chapple, 2020; Yudintceva et al., 2020; Zhang et al., 2021). The advantages of using buccal mucosa instead of other donor sites, such as skin, are the fact that the buccal mucosa is a non-keratinised squamous epithelium that lacks hair (Figure 1) and has low associated morbidity and short harvest time.

Tissue-engineered cell sheets of autologous oral epithelial cells alone, or combined with other tissues have been used for ocular diseases treatment, including eyelid and corneal reconstructions (Nishida et al., 2004; Yoshizawa et al., 2004; Oliva et al., 2020; Sasaki et al., 2020). Using an *in vivo* rabbit model of total limbal SC deficiency, dental pulp SCs were used in a tissue-engineered cell sheet for ocular surface reconstruction (Gomes et al., 2010) highlighting the plasticity of these cells. Furthermore, oral mucosal fibroblasts containing neural crest-origin cells showed plasticity in differentiating into mesenchymal and neural cell lineages, being proposed as an autologous cell source for establishing human corneal epithelial cell sheets suitable for corneal regeneration (Higa et al., 2017). Other applications of the oral mucosa comprise the treatment of tracheal defects (Delaere et al., 2001; Li et al., 2019) and prevention of intrauterine adhesions caused by endometrial damage (Kuramoto et al., 2015).

While in full-thickness skin transplant into the oral cavity (to reconstruct tongue or buccal mucosa), the recipient organ keeps characteristics of the donor tissue (Vural and Suen, 2000; Sebastian et al., 2008; Amin et al., 2011; Aslam-Pervez et al., 2018), there is mounting evidence that grafted epithelial cells can adopt the phenotype of the recipient tissue, stressing the plasticity of these cells and the importance of the underlying connective tissue and fibroblasts to determine epithelial cells phenotype. Recent animal work has successfully grafted oesophageal tissue into skin demonstrating that when exposed to the adult skin dermis, oesophageal epithelial cells transition to a skin identity following a cell fate conversion process (Bejar et al., 2021). This highlights how the stromal cells influence the final epithelial phenotype in a homeostatic tissue. However, this is a topic of much controversy and variability of results over the years, especially when considering clinical and *in vivo* applications (Billingham and Silvers, 1967; Mackenzie and Hill, 1984; Luca et al., 1990; Katou et al., 2003). Whether the epithelium is transplanted alone or with subepithelial tissue as full thickness flaps, or applied as single-cell suspensions of fully differentiated cells or stem cells alone might contribute for the observed heterogenous responses. Additionally, the majority of these events lack a more in depth study of the molecular mechanisms driving the final outcomes. Nonetheless, we can’t discard the intrinsic identity and programmes that epithelial and fibroblastic cells carry themselves, which differ between oral mucosa and skin (Turabelidze et al., 2014; Rinkevich et al., 2015; Iglesias-Bartolome et al., 2018). This heterogeneity could as well explain the predisposition of a tissue to resemble the origin characteristics or to be more influenced by the recipient. Regardless, the studies presented in this final chapter highlight a very promising venue for using these intrinsic cellular properties into other tissue wounds, mainly through improved *in vitro* tissue engineering.

## Conclusion

Techniques to improve skin wound healing are currently under development and are an unmet need, particularly following large burns and war injuries, where treatment is still primarily performed by split-thickness skin grafting and accompanied by problems associated with limited donor tissue, pain and scarring (Singh et al., 2015; Connolly et al., 2016).

Mounting evidence over the last two decades has demonstrated a remarkable plasticity in adult epithelial cell fate while in in different niches, leaving behind the concept of strict SC fates (Bonfanti et al., 2010; Blanpain and Fuchs, 2014; Chacón-Martínez et al., 2018; Yuan et al., 2019). While epithelial cells and fibroblasts reciprocal grafting have dissected their contribution to wound healing, the plasticity of these cells in the new microenvironment and their cellular behaviour need further investigation. There is an exciting future for collaborative efforts to understand the heterogeneity and plasticity between these tissues and yet their common features and the mechanisms behind the cell fate conversion, in order to improve the use of heterotypic transplants for future therapeutic strategies.

In this review, we elucidate the intrinsic properties that prime the oral mucosa with scarless wound healing response. Lessons should be learned from the oral mucosa to apply on other tissues, exploring these unique properties in future innovative therapies combining cell therapy with bioengineering to prevent pathologies associated with impaired wound healing and scar formation in both skin and oesophagus.

## Author Contributions

DP and IS: conceptualisation, writing, editing, and figures. Both authors contributed to the article and approved the submitted version.

## Conflict of Interest

IS is a consultant for L’Oréal Research and Innovation. The remaining author declares that the research was conducted in the absence of any commercial or financial relationships that could be construed as a potential conflict of interest.

## Publisher’s Note

All claims expressed in this article are solely those of the authors and do not necessarily represent those of their affiliated organizations, or those of the publisher, the editors and the reviewers. Any product that may be evaluated in this article, or claim that may be made by its manufacturer, is not guaranteed or endorsed by the publisher.
